# Cotton boll localization method based on point annotation and multi-scale fusion

**DOI:** 10.3389/fpls.2022.960592

**Published:** 2022-08-18

**Authors:** Ming Sun, Yanan Li, Yang Qi, Huabing Zhou, LongXing Tian

**Affiliations:** ^1^School of Computer Science and Engineering, School of Artificial Intelligence, Wuhan Institute of Technology, Wuhan, China; ^2^Hubei Key Laboratory of Intelligent Robot, Wuhan Institute of Technology, Wuhan, China

**Keywords:** deep learning, point annotation, multi-scale, cotton boll, localization

## Abstract

Cotton is an important source of fiber. The precise and intelligent management of cotton fields is the top priority of cotton production. Many intelligent management methods of cotton fields are inseparable from cotton boll localization, such as automated cotton picking, sustainable boll pest control, boll maturity analysis, and yield estimation. At present, object detection methods are widely used for crop localization. However, object detection methods require relatively expensive bounding box annotations for supervised learning, and some non-object regions are inevitably included in the annotated bounding boxes. The features of these non-object regions may cause misjudgment by the network model. Unlike bounding box annotations, point annotations are less expensive to label and the annotated points are only likely to belong to the object. Considering these advantages of point annotation, a point annotation-based multi-scale cotton boll localization method is proposed, called MCBLNet. It is mainly composed of scene encoding for feature extraction, location decoding for localization prediction and localization map fusion for multi-scale information association. To evaluate the robustness and accuracy of MCBLNet, we conduct experiments on our constructed cotton boll localization (CBL) dataset (300 in-field cotton boll images). Experimental results demonstrate that MCBLNet method improves by 49.4% average precision on CBL dataset compared with typically point-based localization state-of-the-arts. Additionally, MCBLNet method outperforms or at least comparable with common object detection methods.

## 1. Introduction

Cotton is a kind of important economic crops in China, as well as important source of fiber and feed. With the increasing demand for sustainable development in modern agriculture (Dubey et al., 2021), cotton production has changed from high yield at any cost to high quality at low cost with better ecological sustainability. Throughout the growth cycle of cotton, cotton bolls are susceptible to pests and diseases such as bollworm and boll rot diseases. In recent years, large-scale fertilization and pesticide spraying are highly required to reduce the impact of pests and diseases on the yield and quality of cotton (Hafeez et al., 2022). A typical example is that cotton bolls are susceptible to verticillium wilt, resulting in premature senescence. Usually spraying a large amount of fungicides on the foliage can prevent the occurrence of Verticillium wilt (Lang et al., 2012). However, this strategy not only requires a mass of labor and material costs, but easily damages the ecological environment of cotton fields (Chi et al., 2021). To save costs and achieve sustainable planting, fixed-point quantitative fertilization and precise pesticide application can be used for misuse and overuse of chemical fertilizer and pesticide. The automatic cotton boll localization method is a key step to realize the precise and intelligent management of cotton fields. In addition, agricultural automation methods such as automatic cotton picking, cotton boll maturity analysis, and yield estimation are also inseparable from cotton boll localization. Therefore, it is necessary to develop a simple, effective and low-cost method for automatic localization of cotton bolls with computer vision technology, which also contributes to the realization of cost saving, quality improvement and sustainable intelligent planting.

The development of computer vision technology has promoted the agricultural automation level. At present, some researchers have studied the usage of image segmentation or object detection methods to automatically identify crop such as apples (Si et al., 2015), tea leaves (Chen and Chen, 2020), grapes (Luo et al., 2016) and cotton (Bhattacharya et al., 2013; Kumar et al., 2016; Singh et al., 2021). These methods usually require bounding box annotations or even pixel-level annotations for supervised learning. Bounding box annotation not only requires high annotation cost, but also inevitably contains some non-target regions, which may allow the model to learn some non-target features and cause misjudgment. Unlike bounding box annotation, point annotation has a relatively low labeling cost and the labeled points must belong to the object. Therefore, it seems possible to explore a simple and robust method for in-field crop localization based on point annotations.

Considering the advantage that point annotation will provide target location information simply and efficiently, a multi-scale cotton boll localization method is proposed based on point annotation and encoder-decoder network structure, named MCBLNet. It is mainly composed of scene encoding for feature extraction and generation of features at different scales, location decoding for location prediction and generation of multi-scale localization maps, and localization map fusion for multi-scale information association. Experiments are conducted to verify the effectiveness of MCBLNet and report relatively accurate localization performance. In general, the proposed MCBLNet method aims to locate cotton boll in real scenes simply and efficiently, and provides a theoretical basis for the realization of sustainable intelligent planting.

## 2. Related work

At present, crop localization methods based on deep learning technology are usually implemented by object detection or segmentation (Agrawal et al., 2016; Su et al., 2021; Franchetti and Pirri, 2022). Among them, the object detection method can be divided into one-stage and two-stage. Typical one-stage object detection methods include SSD (Liu et al., 2016) and YOLO series (Redmon et al., 2016; Redmon and Farhadi, 2017, 2018). Shi et al. (2020) designs channel and spatial masks based on the YOLOv3-tiny network to detect convolution kernels in the network that are closely related to specific target outputs, resulting in more efficient mango detection. Jintasuttisak et al. (2022) used the YOLOv5-m network to detect crowded date palms in UAV images. A series of networks from RCNN (Girshick et al., 2014) to Faster RCNN (Ren et al., 2017) are typical two-stage methods in object detection. Li et al. (2021) adopted a high-resolution network as the backbone to improve Faster RCNN to detect dense hydroponic lettuce seedlings. Mask RCNN (He et al., 2017) is an image segmentation method in the RCNN series of networks. Wang and He (2022) integrated the attention module into the Mask RCNN model, which enhanced the feature extraction ability of the model, thereby segmenting apples of different maturity levels. Although both object detection methods and image segmentation methods can localize crops with little scale variation and high color distinguishability, they require high labeling costs as a basis. Furthermore, both of them may have difficulty effectively detecting small objects in dense images due to the loss of spatial and detailed feature information (Wang et al., 2022). In addition, since objects in highly dense images may overlap each other, the prediction boxes of object detection methods also overlap each other. This will lead to unfriendly visualization results.

Some researchers localize dense objects based on point annotations. Song et al. (2021) proposed the P2PNet network to directly predict a set of points and perform one-to-one matching for dense object localization. Zand et al. (2022) proposed a multi-task dense object localization method based on VGG network. Although these methods can localize dense objects only with point annotations, they are difficult to localize various crops effectively due to their limited feature extraction ability, field crop scale changes, and natural plant growth changes. In addition, Ronneberger et al. (2015) proposed a fully connected network UNet based on the encoder-decoder structure, which can effectively extract image features while using skip connections to further enhance the localization accuracy. Ribera et al. (2019) located different targets in different scenes based on UNet. To sum up, a simple and efficient cotton boll location method based on point annotation may be designed by combining the dense crowd location strategy based on point annotation with the structure of UNet network.

## 3. Materials and methods

### 3.1. Materials

**Figure 1:**
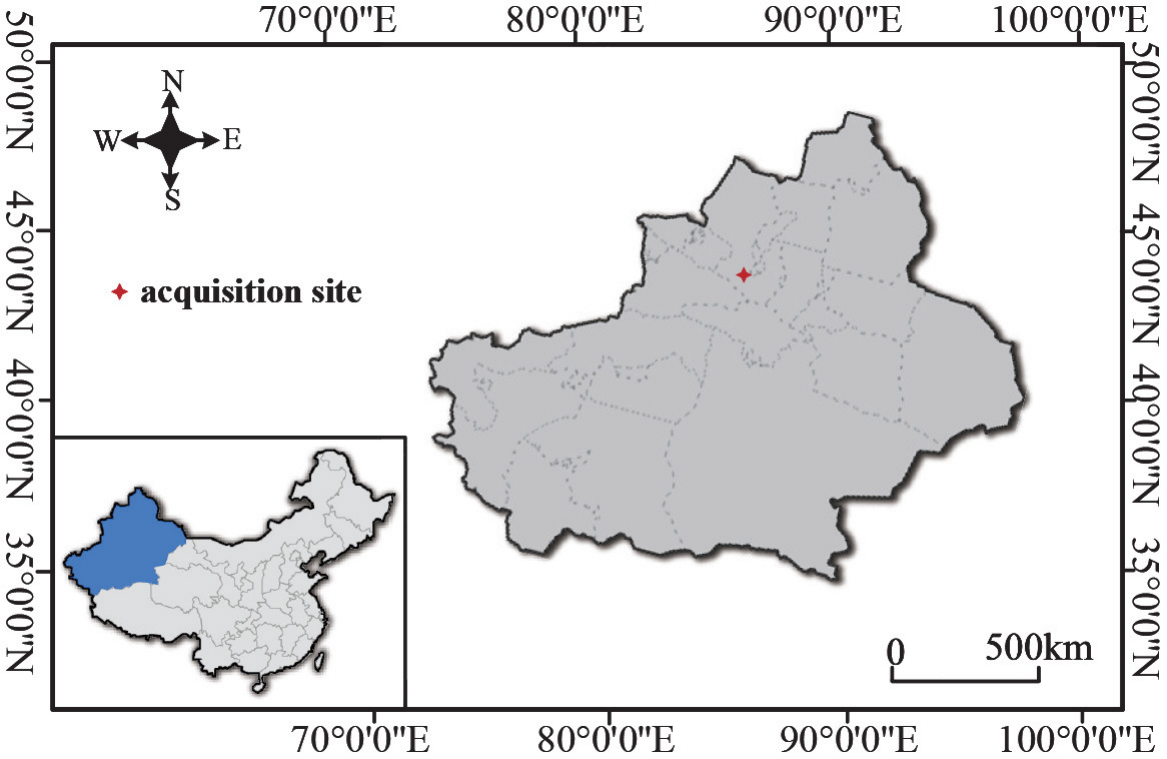
Location of field boll image acquisition sites.

The CBL dataset targets in-field cotton boll localization. Field cotton boll images were collected in Xinjiang Uygur Autonomous Region (44.18N, 86E), and taken under natural illumination by the ground-based non-contact observation system (Li et al., 2020). The geographical location of the image acquisition is shown in Figure 1. Three hundred images exposed normal images were selected from images collected from 2016 to July and August 2018 to compose the CBL dataset, which contains a series of cotton field images during the growth cycle of cotton bolls.

**Figure 2:**
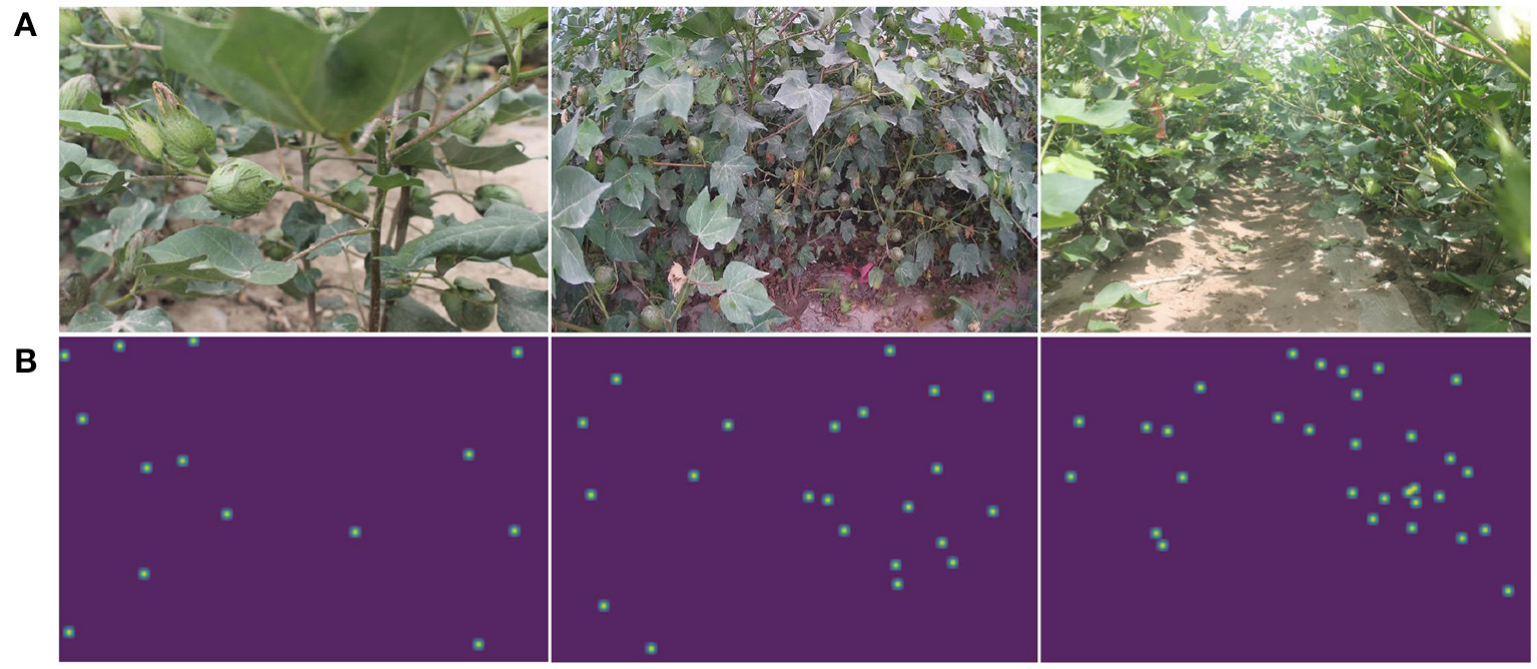
Example of CBL dataset. **(A)** Is the original image, **(B)** is the corresponding ground-truth.

As shown in Figure 2, the dataset consists of front view images of field bolls with four different resolutions of 3088 × 2056, 3456 × 2304, 1920 × 1080, and 5184 × 3456. In order to ensure the diversity of cotton boll images during the experiment, 180 images are used for training, 58 images are used for validation, and the other 62 images are used for testing. Following standard practice (Lu et al., 2021), the center of each boll is manually annotated with a point since point annotations provide information on the location and class of the target. The number of cotton bolls in the image varies from 1 to 44, and a total of 5,794 boll instances were finally annotated. Ground-truth is generated by Gaussian smoothing on a matrix of annotated points. The labeling tool used is LabelMe, which can be found at https://github.com/wkentaro/labelme.

### 3.2. Methods

**Figure 3:**
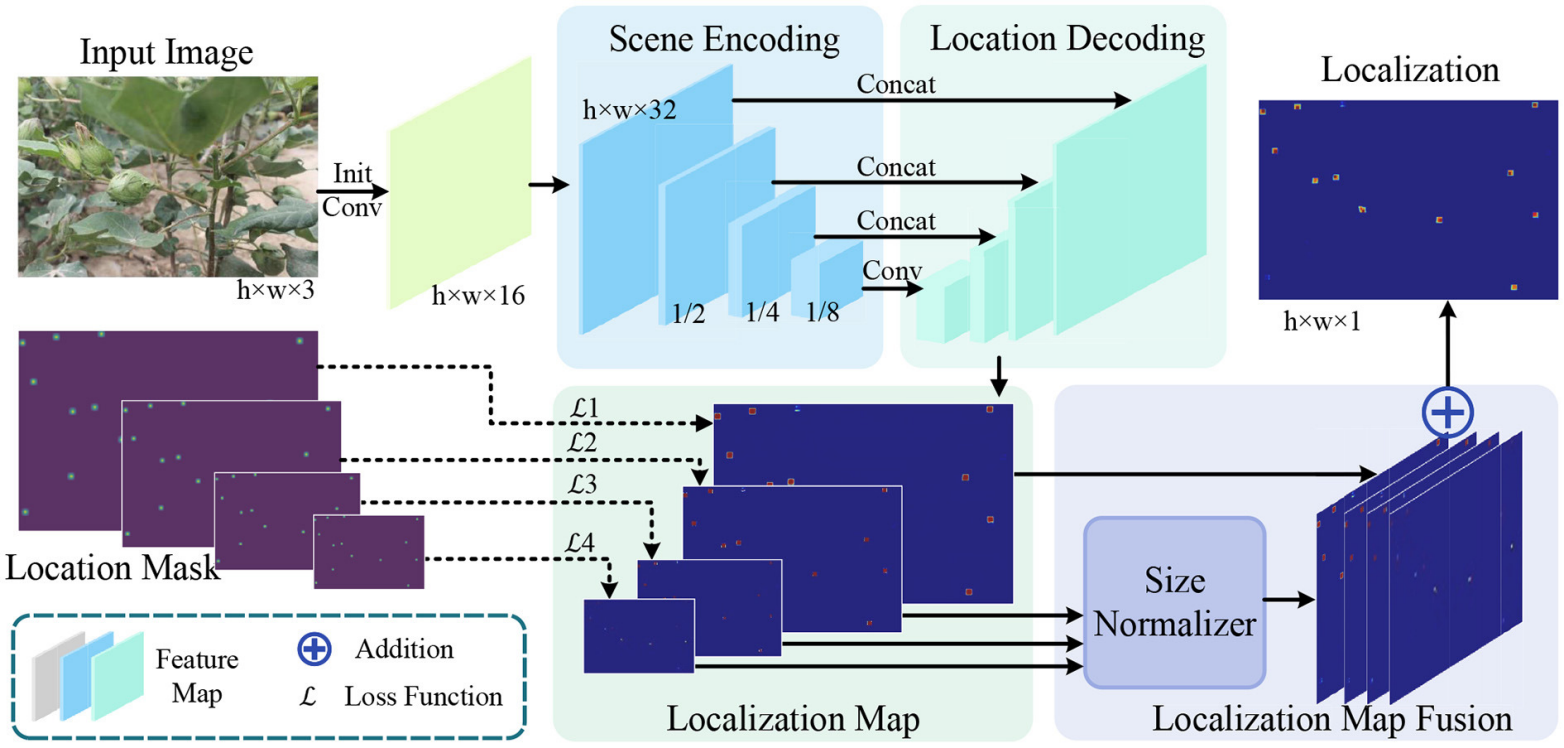
The pipeline of MCBLNet network architecture.

In this section, we introduce our proposed fully convolutional network MCBLNet based on UNet. MCBLNet aims to learn a mapping from an input image of size *h* × *w* × 3 to a localization map of size *h* × *w* × 1, as shown in Figure 3. MCBLNet mainly composed of scene encoding for feature extraction, location decoding for position prediction and localization map fusion for multi-scale information association. The scene encoding, location decoding, and localization map fusion are as follows.

#### 3.2.1. Scene encoding and location decoding

**Figure 4:**
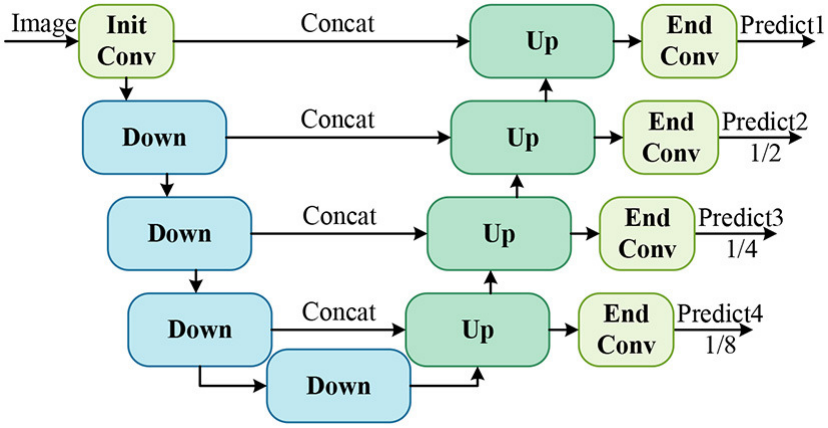
Detailed structure of scene encoder and location decoder.

Referring to the UNet network (Ronneberger et al., 2015), the detailed structure of feature encoding and multi-scale localization map prediction in MCBLNet is shown in Figure 4. It consists of an initial convolution block, four Down modules, four UP modules, and four end convolution blocks. The initial convolution (Init Conv) block is a set of two 3 × 3 convolutions with a stride of 1 and a padding of 1 for channel number expansion; the end convolution (End Conv) block is a combination of 1 × 1 convolution and sigmoid function for localization map prediction; Down module for scene encoding; Up module for location decoding. Scene encoding and localization decoding map the input image of size *h* × *w* × 3 into four localization maps of size *h* × *w* × 1, $\frac{h}{2}\times \frac{w}{2}\times 1$, $\frac{h}{4}\times \frac{w}{4}\times 1$, and $\frac{h}{8}\times \frac{w}{8}\times 1$, respectively. The four localization maps are designed to locate cotton bolls at different scales to ultimately reduce the missed detection rate.

**Figure 5:**
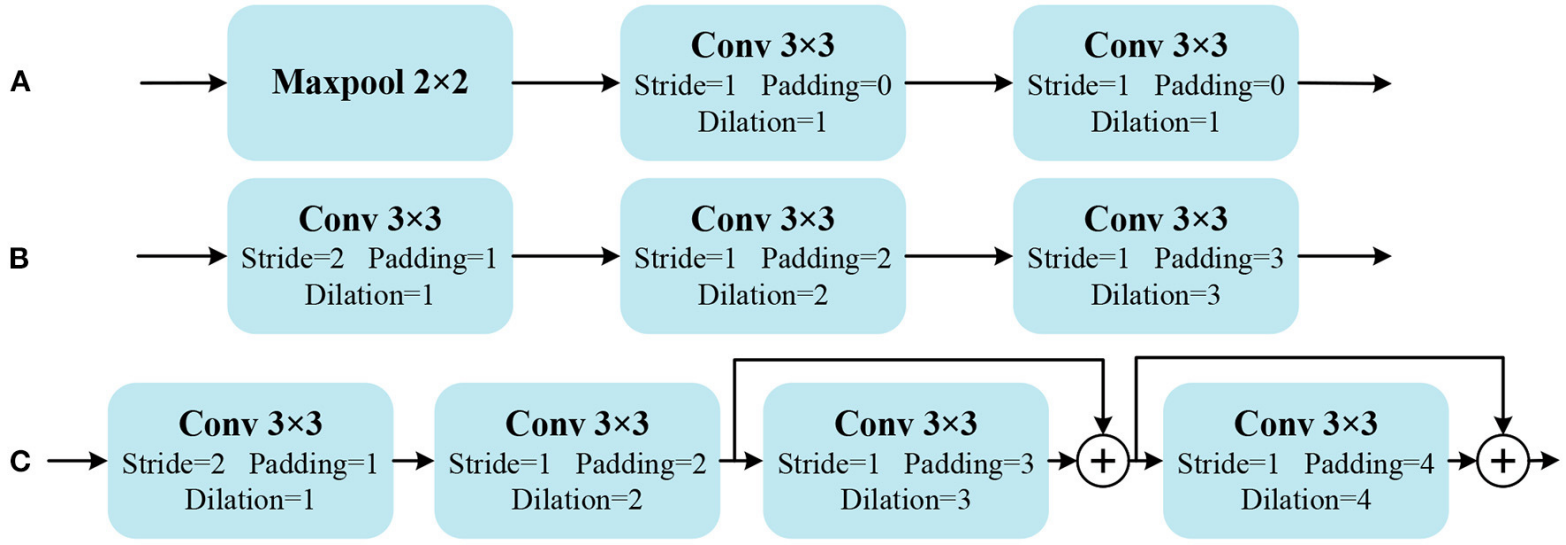
Down module structures. **(A)** Is Down of UNet, **(B)** is Down of MCBLNet-lite, **(C)** is Down of MCBLNet. “+” indicating matrix addition.

Due to the repetitiveness of the structure, a part of the continuous downsampling convolution process in the UNet network is defined as the Down module, and its structure is shown in Figure 5A. It consists of a 2 × 2 max pooling layer for dimensionality reduction and two 3 × 3 convolutions with stride 1. Researchers have demonstrated that pooling layers can cause drastic changes in the output (Zhang, 2019). To obtain stable image features, the pooling layer is replaced by strided convolution. In order to expand the receptive field, the original ordinary convolution is replaced by three dilated convolutions with gradually increasing dilation rates. Then a new Down module is constructed as shown in Figure 5B. The network structure constructed by Down module is called MCBLNet-lite. In addition, to further enhance the feature extraction capability of the network, a 3 × 3 dilated convolution with a dilation rate of 4 is added. At the same time, skip connections are added to further utilize redundant information. Then the final Down structure is constructed as shown in Figure 5C. The final Down module is adopted in the proposed MCBLNet network.

**Figure 6:**
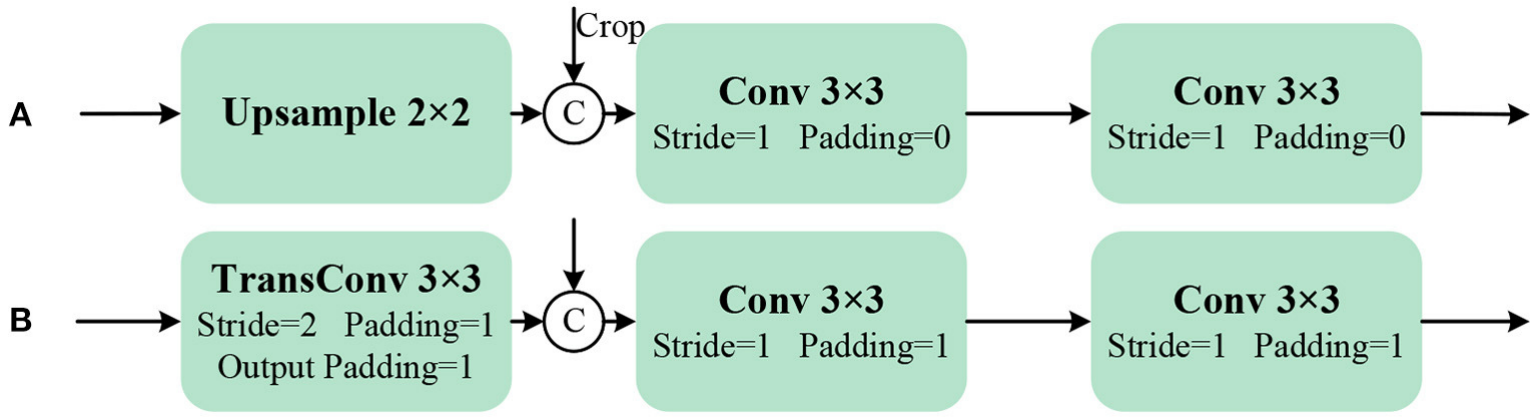
Up module structures. **(A)** Is Up of UNet, **(B)** Is Up of MCBLNet. “C” representing concatenate.

Similar to the Down module, a part of the continuous upsampling convolution process in the UNet network is defined as the Up module, shown in Figure 6A. In order to obtain more stable localization results, the upsampling layer is replaced with a transposed convolution (TransConv) for trainable upsampling. A new Up module is constructed as shown in Figure 6B.

The convolutions in the UNet network are not padded, so the output localization map is smaller than the original input image. Therefore, it needs to be cropped before performing the concatenate operation. In order to simplify the operation and improve the robustness of the model, the corresponding padding is set for the convolution in MCBLNet, so that the size of the feature map after each layer of convolution is fixed. The output localization map size of MCBLNet is fixed to 1, $\frac{1}{2}$, $\frac{1}{4}$, and $\frac{1}{8}$ times the input image. In fact, the output localization map is a fixed-size 2D matrix. The value of each point in the matrix represents the probability that this point is predicted to be the target. So the location map can be expressed as:


(1) $$\begin{array}{r} M_{L}={\left[p_{j}^{i}\right]}_{m}^{n},0<i<n,0<j<m \end{array}$$

Among them, ${\left[*\right]}_{m}^{n}$ represents a matrix of size *n*×*m*, and $p_{j}^{i}$ represents the probability that the point in the *i*th row and the *j*th column is the target.

#### 3.2.2. Localization map fusion

After scene encoding and localization decoding, the original input image is mapped into 4 predicted localization maps of different sizes. To obtain uniform and accurate localization results, it is necessary to combine the results of the four localization maps. Generally speaking, small-sized localization maps are more robust to large targets, and large-sized localization maps are more robust to small targets. In order to reduce the missed detection rate and false detection rate, localization map fusion module is designed to fuse four different localization maps to obtain the final localization map. First, the size normalizer in Figure 3 upsamples the localization maps with sizes of $\frac{1}{8}$, $\frac{1}{4}$, and $\frac{1}{2}$, respectively to the same size as the original image. The upsampled 4 localization maps are added to obtain the final predicted localization map with the same size as the original input image. Then the final predicted location map can be expressed as:


(2) $$\begin{array}{r} FM_{L}={\left[\sum_{k=1}^{4}{\left(p_{k}\right)}_{j}^{i}\right]}_{w}^{h} \end{array}$$

where *h* and *w* are the height and width of the original image, respectively.

#### 3.2.3. Loss function

Each point in the four predicted localization maps obtained by the proposed method represents the probability of whether the store belongs to the target. The cross-entropy loss function mainly describes the distance between actual output probability and expected output probability (Farahnak-Ghazani and Baghshah, 2016). Therefore, the cross-entropy loss function can be used to calculate the distance between each point in the predicted location map and the corresponding point in the ground-truth, which is expressed as:


(3) $$\begin{array}{r} L_{BCE}=-\overset{N}{\underset{i=1}{\sum }}{\hat{p}}_{i}logp_{i} \end{array}$$

where *N* is the number of pixels in the image, *p*_*i*_ is the probability that the model predicts the *i*th pixel as a positive sample, and ${\hat{p}}_{i}$ is the true value of the *i*th pixel.

To accurately localize each object at each scale, the cross-entropy loss for each scale is computed separately. The total loss at final training is the sum of the losses generated from aforementioned four different scale maps, which can be expressed as $L_{total}=\overset{4}{\underset{k=1}{\sum }}L_{k}$.

## 4. Results and discussion

### 4.1. Implementation details

In order to reduce the amount of model parameters as much as possible without reducing the location accuracy, the number of output channels from the initial convolution block to the scene encoder is 16, 32, 128, 256, and 512 in turn. The number of output channels of the location decoder is 256, 128, 64, and 32 in sequence. In training, the resolution of images is resized to 768 × 512 to enable batch training without excessively missing the target pixels of the boll. Inspired by Ronneberger et al. (2015), the 768 × 512 input image is cropped into 12 image patches (256 × 256) for training separately to speed up training and perform data augmentation. The parameters epoch, batch size, and learning rate are set to 60, 16, and 0.0001, respectively.

Our method is implemented based on pytorch. All experiments are implemented on a server with Intel Core i9-10900X CPU at 3.70GHz and GeForce RTX 3090. The software is Ubuntu20.4 and python3.6.

### 4.2. Evaluation metrics

The object localization performance of the MCBLNet is evaluated by average precision (AP) (Everingham et al., 2015), a commonly used evaluation metric for object detection methods to ensure fairness and accuracy. AP is the area under the precision (P) and recall (R) curves. The calculation methods of P, R and AP are:


(4) $$\begin{array}{r} P=\frac{TP}{TP+FP} \end{array}$$

(5) $$\begin{array}{r} R=\frac{TP}{TP+FN} \end{array}$$

(6) $$\begin{array}{r} AP=\int_{0}^{1}P\left(R\right)dR \end{array}$$

where TP (true positive) is the number of correct localizations in all targets, FP (false positive) is the number of incorrect localizations in all targets, and FN (false negative) is the number of targets that were not detected. The connected domain of all points with predicted probability exceeding 50% is regarded as the predicted localization area. The set of their center points is used as the prediction point set. The nearest neighbor distance between prediction point set and ground-truth point set is calculated as the evaluation condition. Similar to Ribera et al. (2019) and Zand et al. (2022), the point is considered to belong to TP when the predicted point is within 10 pixels of some ground-truth point, otherwise it is classified to FP. Each ground-truth point is matched against only one predicted point. The APs used later are all AP50s.

In addition, FPS and parameter amount (Param) are used to evaluate the running speed and storage cost of the model. FPS represent the number of images that can be detected per second, and the size of the parameter amount refers to the size of space occupied by the model.

### 4.3. Model evaluation

#### 4.3.1. Comparison of different localization methods

To demonstrate the effectiveness of the proposed method for cotton boll localization, we compare it with several object localization networks and object detection networks on the CBL dataset. Specifically, comparisons are made with the bounding box annotation-based SSD (Liu et al., 2016), FasterRCNN (Ren et al., 2017), YOLOv3 series (Redmon and Farhadi, 2018), and YOLOv5 series and point annotation based object localization methods P2PNet (Song et al., 2021) and MSPSNet (Zand et al., 2022). The specific experimental results are shown in Table 1.

**Table 1:** Table of experimental results for each method on the CBL dataset.

**Model**	**Label**	**AP(%)**	**FPS**	**Param(M)**
SSD	Box	8.23	13.18	95
Faster RCNN	Box	38.5	9.67	165.7
YOLOv3-tiny	Box	51.1	**33.34**	17.4
YOLOv3-spp	Box	64.4	28.21	125.6
YOLOv5m	Box	60.8	28.92	42.2
YOLOv5s	Box	57.2	31.42	**14.4**
P2PNet	Point	8.3	23.38	86.4
MSPSNet	Point	34.5	7.16	263.3
MCBLNet-lite	Point	78.3	22.5	37.8
MCBLNet	Point	**83.9**	20.86	50.3

*The optimal values in each column are bold-faced*.

The localization performance of MCBLNet-lite and MCBLNet methods on the CBL dataset is better than other compared methods, as shown in Table 1. Specifically, the AP of the MCBLNet is improved by 49.4% compared with the best point-based target localization algorithm MSPSNet, and the model parameter amount is only one-fifth of that. Compared with the best bounding box annotation based object detection algorithm yolov3-spp, the point annotation based MCBLNet method has an AP improvement of 19.5% with comparable detection speed.

#### 4.3.2. Comparative experiments under different density distributions

**Figure 7:**
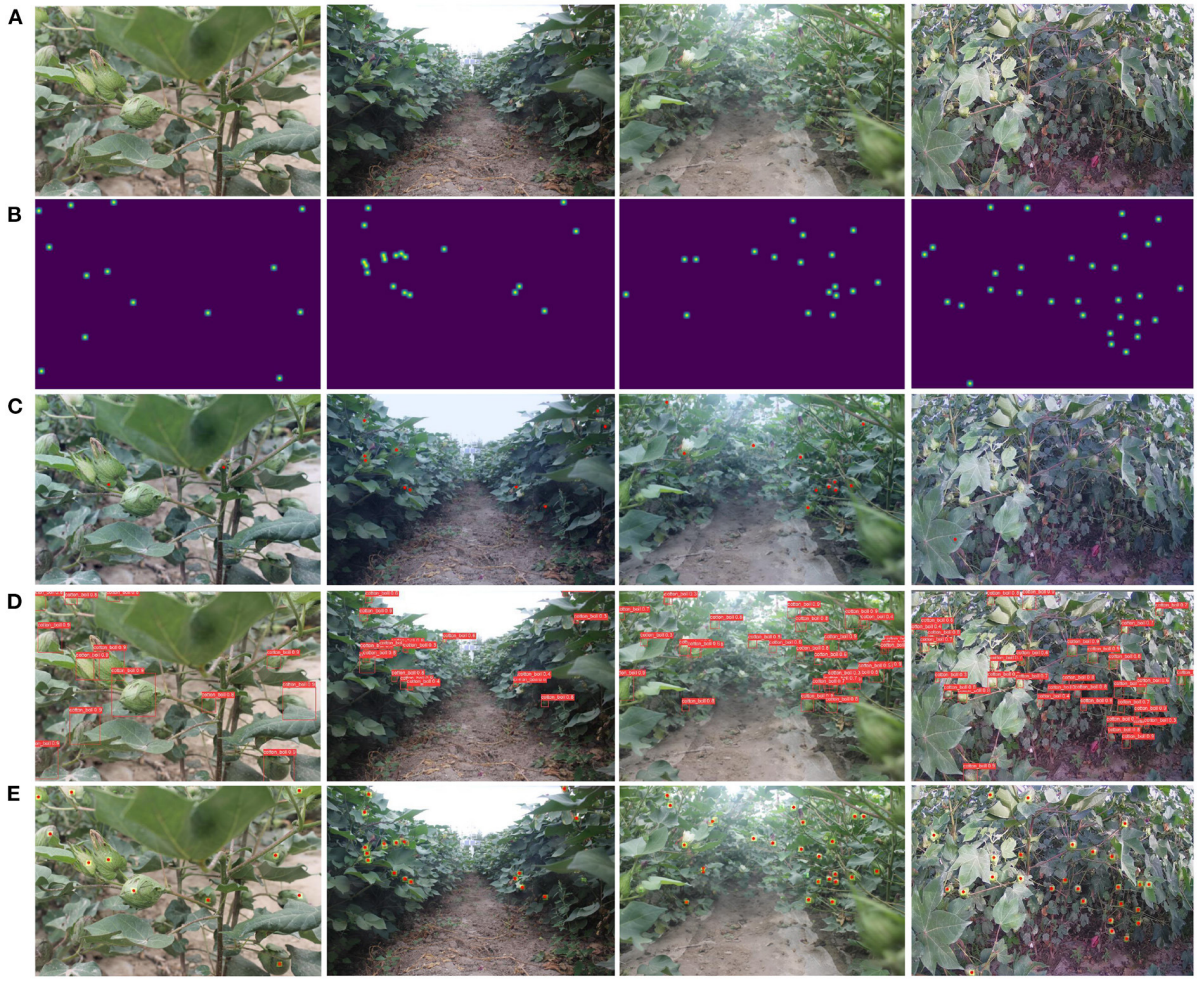
Localization effect of three methods in different density images. **(A)** Is original image and **(B)** is ground-truth. **(C–E)** Are the localization effects of MSPSNet, YOLOv3-spp, and MCBLNet, respectively.

The accuracy of the model may be affected by different occlusions and cotton boll counts in images of different densities distributions. Contrastive experiments are carried out according to the difference of object density in the CBL test images. Referring to the settings of Wang et al. (2022), images containing 10-20 cotton bolls are considered as moderately dense, and images containing more than 20 cotton bolls are considered as highly dense. Experiments are conducted on moderately dense and highly dense images with YOLOv3-spp, MSPSNet, and MCBLNet, respectively. Among them, YOLOv3-spp is the best localization method based on bounding box annotation in Table 1, and MSPSNet is relatively better among the localization methods based on point annotation except MCBLNet. The experimental results are shown in Table 2 and Figure 7.

**Table 2:** Localization results of three methods under different density distributions.

**Model**	**Moderately dense**			**Highly dense**		
	**P (%)**	**R (%)**	**AP (%)**	**P (%)**	**R (%)**	**AP (%)**
MSPSNet	74.7	18.2	14.3	78	16.9	34.3
YOLOv3-spp	74.5	68.4	67.6	75.7	59.3	63.7
MCBLNet	69	56.8	61.7	82.9	58.6	83.9

It can be seen from Table 2 that the object detection method based on bounding box annotation has better accuracy for moderately dense cotton boll images than highly dense cotton boll images. The localization method based on point annotation is more accurate for highly dense cotton boll images than for moderately dense cotton boll images. It indicated that the localization method based on point annotations is more robust in localizing dense objects. Specifically, MCBLNet achieves 83.9% AP for high-density cotton boll images and comparable AP to YOLOv3-spp for moderately dense cotton boll images. It demonstrated that MCBLNet has better localization performance for cotton bolls with different densities.

The localization effect is shown in Figure 7. The red dots in Figure 7C are the predicted anchor points by MSPSNet. The yellow-green blob in Figure 7E is the prediction area by MCBLNet, and the red point is the center of blob. Compared with YOLOv3-spp and MCBLNet, MSPSNet has a large number of missed detections. When detecting dense boll regions, some prediction boxes of YOLOv3-spp overlap each other. Contrary to YOLOv3-spp, the prediction points of MCBLNet are distinguishable. Therefore, compared with YOLOv3-spp, MCBLNet has better visual localization results.

#### 4.3.3. Ablation study

To measure the contribution of various factors to MCBLNet, ablation experiments are performed on the CBL dataset. The experimental results are shown in Table 3, in which Enhance Down represents the final Down module, and Map Fusion means the localization map fusion module.

**Table 3:** Ablation experiments on the CBL dataset.

**Model**	**Configurations**		**AP (%)**	**FPS**	**Param(M)**

	**Enhance**	**Map**			
	**down**	**fusion**			
MCBLNet-lite_base			71.8	22.5	37.8
MCBLNet-lite		✓	78.3	22.5	37.8
MCBLNet_base	✓		82.4	20.86	50.3
MCBLNet	✓	✓	83.9	20.86	50.3

“*✓” means joining the corresponding module*.

Comparing the experimental results of MCBLNet-lite and MCBLNet, the Enhance Down module can enhance the feature extraction ability by increasing the number of parameters. The AP of MCBLNet-lite is 6.5% higher than that of MCBLNet-lite_base, and the AP of MCBLNet is 1.5% higher than that of MCBNet_base. It can be seen that the localization map fusion module can improve the AP without increasing the amount of parameters and without affecting the running speed.

## 5. Conclusion

In this paper, a point annotation-based cotton boll localization method named MCBLNet is proposed. It can solve the localization problem of multi-scale objects in complex backgrounds simply and efficiently. The method mainly includes three parts: scene encoding which can effectively extract image features, location decoding which can output multi-scale localization maps and localization map fusion which can combine localization map information of different scales. Experiments were conducted on the CBL dataset. Experimental results show that the localization performance of our method significantly outperforms other point-annotation-based localization methods, and the performance is also better than or at least comparable to bounding-box annotation-based localization methods. Overall, the MCBLNet can simply and robustly locate crops using only point annotations.

In future work, we consider to fundamentally solve the problem of insufficient target feature extraction by further combining the structural characteristics of corresponding cotton boll to optimize the labeling method. At the same time, we also plan to add some output headers to reuse the extracted target features for object counting. In addition, location methods can be used in some practical agricultural applications, such as directional high-efficiency water-saving irrigation, fixed-point quantitative fertilization and precision pesticide application.

## Data availability statement

The raw data supporting the conclusions of this article will be made available by the authors, without undue reservation.

## Author contributions

MS and YL designed the experiments and wrote the manuscript. MS and YQ performed the experiments. YL provided the hardware and software support and reviewed and edited the manuscript. YL and HZ participated in project management, provided the resources, and contributed to funding. MS, YQ, and LT contributed to experiments and algorithmic application. All authors have read and agree to the published version of the manuscript.

## Funding

This work was supported in part by National Natural Science Foundation of China under Grants 61906139 and 62171327, in part by Knowledge Innovation Program of Wuhan-Shuguang Project under Grant 2022010801020359, in part by Science Foundation of Wuhan Institute of Technology under Grant K202031, in part by the Hubei Key Laboratory of Intelligent Robot (Wuhan Institute of Technology) of China under Grant HBIRL 202108, and in part by Graduate Innovative Fund of Wuhan Institute of Technology under Grant CX2021257.

## Conflict of interest

The authors declare that the research was conducted in the absence of any commercial or financial relationships that could be construed as a potential conflict of interest.

## Publisher's note

All claims expressed in this article are solely those of the authors and do not necessarily represent those of their affiliated organizations, or those of the publisher, the editors and the reviewers. Any product that may be evaluated in this article, or claim that may be made by its manufacturer, is not guaranteed or endorsed by the publisher.
