# Corrigendum: Cotton boll localization method based on point annotation and multi-scale fusion

**DOI:** 10.3389/fpls.2023.1213003

**Published:** 2023-05-30

**Authors:** Ming Sun, Yanan Li, Yang Qi, Huabing Zhou, LongXing Tian

**Affiliations:** ^1^ School of Computer Science and Engineering, School of Artificial Intelligence, Wuhan Institute of Technology, Wuhan, China; ^2^ Hubei Key Laboratory of Intelligent Robot, Wuhan Institute of Technology, Wuhan, China

**Keywords:** deep learning, point annotation, multi-scale, cotton boll, localization

In the published article, there was an error in the **Funding statement**. The funding statement for the Knowledge Innovation Program of Wuhan-Shuguang Project was displayed as “2022010801020350”. The correct statement is “Knowledge Innovation Program of Wuhan-Shuguang Project (2022010801020359). ”

The correct Funding statement appears below.

“This work was supported in part by National Natural Science Foundation of China under Grants 61906139 and 62171327, in part by Knowledge Innovation Program of Wuhan-Shuguang Project under Grant 2022010801020359, in part by Science Foundation of Wuhan Institute of Technology under Grant K202031, in part by the Hubei Key Laboratory of Intelligent Robot (Wuhan Institute of Technology) of China under Grant HBIRL 202108, and in part by Graduate Innovative Fund of Wuhan Institute of Technology under Grant CX2021257.”

The authors apologize for this error and state that this does not change the scientific conclusions of the article in any way. The original article has been updated.

